# Optimization of Uruset Apple Vinegar Production Using Response Surface Methodology for the Enhanced Extraction of Bioactive Substances

**DOI:** 10.3390/foods8030107

**Published:** 2019-03-22

**Authors:** Seydi Yıkmış

**Affiliations:** Department of Nutrition and Dietetics, Tekirdağ Namık Kemal University, Değirmenaltı 59030 Tekirdağ, Turkey; syikmis@nku.edu.tr

**Keywords:** uruset apple, ultrasound, total phenolic content, total flavonoid content, response surface methodology

## Abstract

In this study, the aim is to produce non-thermal vinegar by using red Uruset apples, which have high bioavailability among apple varieties. For this purpose, Uruset apple vinegar was produced and ultrasound at different times (2, 4, 6, 8 and 10 min) and different amplitudes (40%, 50%, 60%, 70%, and 80%); in addition, a 26 kHz frequency was applied to the samples. Total phenolic content (TPC), total flavonoid content (TFC), total antioxidant capacity (1,1-diphenyl- 2-picrylhydrazyl (DPPH) and cupric reducing antioxidant capacity (CUPRAC)), and color values were evaluated for the optimization of process conditions. At the same time, the differences between commercial apple vinegar (CV), pasteurized Uruset apple vinegar (PV), and a control (C) of untreated apple vinegar were investigated. Ultrasound treatment of Uruset apple vinegar was more successful for the enrichment of bioactive substances than the other samples. At the end of the study, the maximal optimization values for Uruset apple vinegar were 7.4 min and 62.2 amplitude. At the end of optimization, CUPRAC (0.69 mg TEAC/mL), DPPH (0.49 mg TEAC/mL), total flavonoid content (46.95 mg CE/L), and total phenolic content (124.25 mg GAE/L) were determined. As a result, ultrasound technology was successfully used for Uruset apple vinegar production.

## 1. Introduction

The word vinegar is derived from the French *vin aigre*, meaning “sour wine”; it can be made from almost any product that includes a source of fermentable carbohydrates, including wine, molasses, sorghum, apples, pears, grapes, strawberries, melons, coconut, honey, beer, potatoes, beets, maple syrup, malt, cereal, and whey [1]. Vinegar, which is widely used as a flavoring and preservative in foods, is also a product traditionally used in the treatment of various diseases since ancient times. Thanks to various phenolic compounds, amino acids, vitamins, organic acids, and melanoidins in its contents, vinegar are many beneficial effects on health, especially antimicrobial, antioxidant, antidiabetic, and anticarcinogenic effects. It also reduces the consumption of food, helps weight loss indirectly, and plays an important role in the absorption of calcium, etc. [2,3].

In recent years, there has been an increase in the consumption of vinegar, which is noteworthy for its effects on health. It is reported that the methods used during vinegar production affect its bioactive components, and the functional properties of vinegar produced by conventional methods may be higher than industrial vinegar. Phenolic substances, which change depending on the raw material used and the production method, affect the antioxidant and antimicrobial potential of vinegar [4,5].

Nowadays, with the introduction of new technologies to the world of science, new methods have been developed, and the advantages of these technologies are utilized in current processes. In this context, the trend towards the use of ultrasound and pasteurization has increased recently compared to non-thermal technologies. In studies using ultrasound therapy on liquid foods, significant microbial inactivation was reported, with promising results of minimal effects on the deterioration of quality parameters and improved functionality [6].

Experimental design is required to determine the parameters of processes in experimental studies and to reach the correct analysis results. The mathematical models, which are a function of optimization processes and the resulting variables, play a key role in making predictions about the process before the experiments, from system design and from laboratory-scale studies to industrial systems. Given these reasons, the necessity of optimizing a study is better understood. In general, optimization is the process of bringing together and applying the process by considering the interactions of independent variables, as well as the effects of the independent variables on the target (response) in accordance with the determined objectives (responses). The response surface method, which is one of the optimization methods, is a technique used for high yields and product quality acceptance in food processing, including optimization [7].

Antioxidant-rich components, carbohydrates, essential minerals, and dietary fiber contained in apples have an important place in terms of taste and nutrient content. In the study, the red Uruset diamond variety was used because of its high bioactive components compared to other apple varieties [8,9]. Ultrasound and Uruset apple vinegar provided the opportunity to optimize the vinegars’ ability to ensure safety, without adversely affecting quality and consumer perception and without harming nutrients. Therefore, this study evaluated process optimization by evaluating antioxidants, phenolic compounds, flavonoids, and color properties with ultrasounds of different time and amplitudes. At the same time, the differences between commercially produced apple vinegar, pasteurized Uruset apple vinegar, and untreated Uruset apple vinegar were examined.

## 2. Materials and Methods

### 2.1. Chemicals and Reagents

All the chemicals, reagents, and solvents used in the assay protocols were of analytical grade. Catechin, 1,1-diphenyl- 2-picrylhydrazyl (DPPH), gallic acid, 6-hydroxy2,5,7,8-tetramethylchroman-2-carboxylic acid (Trolox) and Neocuprin were obtained from Sigma Aldrich, Hamburg, Germany. Folin-Ciocalteu reagent, sodium hydroxide, and sodium carbonate were from Merck (Darmstadt, Germany).

### 2.2. Production of Uruset Apple (Cultivar with Red Penetralia) Vinegar

Apple juices (15 °Brix) were obtained from dilutions of 70 °Brix concentrated apple juice. Apple juices were filled into 5 L sterile jars and inoculated with commercial wine yeast (*Saccharomyces cerevisiae*, 0.3%). After the containers were covered with air lock plugs, they were left for the alcohol to ferment for 40 days at 25 °C. Wine (5 L) was transferred into sterile jars and inoculated with sharp vinegar (5%) as a natural acetic acid culture. After the wine, the mixture was fermented for 50 days at 28 °C, until the ethanol content was 0.5% to 1%. The control (C) sample was untreated Uruset vinegar. Pasteurization of bottled Uruset apple vinegars was performed in a water bath (Wisd-Model WUC-D06H, Daihan, Wonju, Korea). Pasteurized Uruset vinegar (PV) was processed at 66 °C for 30 min. Commercial apple vinegar (CV) was supplied from the market. Vinegar samples were stored at 4 °C in 100 mL sterile glass jars for use in analysis. Tests were performed three times.

### 2.3. Ultrasound Treatments

Sonication treatments were performed directly after fresh juice was extracted. Uruset vinegar was treated at 26 kHz frequency at different times (2, 4, 6, 8 and 10 min) and amplitudes (40%, 50%, 60%, 70% and 80%). The sonication was performed at 26 kHz frequency and a temperature of 20 °C, using a 200 W ultrasonic processor (Model UP200St, Hielscher Ultrasonics, Teltow, Germany). All the sonication treatments were carried out in the dark to avoid any possible interference from light. Uruset vinegar samples (sonicated) were kept in sterilized and air-tight media bottles, and were stored at 4 °C until further analysis.

### 2.4. Experimental Design

The Uruset apple vinegar was analyzed using Minitab Statistical Analysis Software (Minitab 18.1.1) to optimize the effect of the ultrasound on quality parameters. The response surface method (RSM) was used. A central composite design was chosen as the experimental design, and a five-level, two-factor experiment design was created. There were 13 test points for optimization (Table 1 and Table 2). Model competence, R**^2^** and corrected -R**^2^** coefficients, lack-of-fit tests, and ANOVA results were evaluated. Arguments were determined as time (*X***_1_**) and amplitude (*X***_2_**). Dependent variables were determined as total phenolic content (TPC), total flavonoid content (TFC), antioxidants (1,1-diphenyl- 2-picrylhydrazyl (DPPH) and cupric reducing antioxidant capacity (CUPRAC)), and color values. The second order polynomial equation, which is shown below, was used to create the model Equation (1):

(1) $$y=\beta_{0}+\sum_{i=1}^{3}\beta_{i}X_{i}+\sum_{i=1}^{3}\beta_{ii}X_{i}^{2}+\sum_{\begin{array}{c} i=1\\ i<j \end{array}}^{3}\sum_{j=1}^{3}\beta_{ij}X_{i}X_{j}$$

where quality *y* is the dependent variable, *β*_0_ is intersection term, *β_i_* is the first-order (linear) equation coefficient, *β_ii_* is the quadratic coefficient of coefficient, *β_ij_* is a two-factor cross-correlation coefficient, and *X_i_* and *X_j_* are independent variables.

### 2.5. Determination of Total Phenolic Content and Total Flavonoid Content 

The total phenolic content was measured spectrophotometrically by the Folin–Ciocalteu method [10]. A vinegar sample of 0.1 mL, with 0.90 mL of distilled water and 5 mL of 0.2 N Folin–Ciocalteau solution (Merck, Germany), was mixed with 4 mL of 7.5% sodium carbonate solution (Merck, Germany). This was incubated for 2 h in the dark at room temperature. The absorbance changes were determined with a spectrophotometer (SP-UV/VIS-300SRB, Spectrum Instruments, Melbourne, Australia) at 765 nm. Gallic acid (Sigma Aldrich, Germany) was used as a reference standard, and the results were expressed as mg gallic acid equivalent per liter of vinegar (mg GAE/L).

The total flavonoid content was modified by the aluminum chloride colorimetric analysis method [11]. An aliquot (1.0 mL) of the vinegar sample was placed in different test tubes containing 4 mL of distilled water, then 0.3 mL of sodium nitrite (5% NaNO**_2_**, *w*/*v*) was added and allowed to stand for 5 min. Later, 0.3 mL of aluminum trichloride (10% AlCl**_3_**.6H**_2_**O) was added and incubated for 5 min, followed by the addition of 2 mL of 1 M sodium hydroxide (NaOH), and the total volume was made up to 10 mL with distilled water. Samples were allowed to incubate in the dark for 30 min. The absorbance changes were determined with a spectrophotometer (SP-UV/VIS-300SRB, Melbourne, Australia) at 510 nm. The TFC was expressed as mg catechin equivalent (CE) per liter.

### 2.6. Determination of Total Antioxidant Activity (DPPH and CUPRAC)

The antioxidant activity of each sample was also estimated using a DPPH radical scavenging assay with slight modifications [12]. To a known aliquot (1 mL) of vinegar, 1 mL of DPPH (1,1-diphenyl 2-picrylhydrazyl) solution (0.2 mM in methanol) was added, followed by incubation in the dark for 30 min at room temperature (25 ± 1 °C). The absorbance changes were determined with a spectrophotometer (SP-UV/VIS-300SRB, Melbourne, Australia) at 517 nm. The results were expressed in mg Trolox equivalent (TEAC)/L.

The antioxidant activity was measured by the CUPRAC (cupric reducing antioxidant capacity) test [13]. Samples of 1 mL of 0.01 M copper chloride (CuCl**_2_**), 1 mL of 7.5 × 10^−3^ Neocuprin (Sigma Aldrich, Germany), 1 mL of 1 M ammonium acetate solution, and 1 mL of purified water was added at 20 °C with incubation for 1 h. The absorbances were determined by a spectrophotometer (SP-UV/VIS-300SRB, Melbourne, Australia) at 450 nm. Calculations were made by using the standard calibration curve prepared with Trolox (Merck, Germany).

### 2.7. Color Analysis

The color coefficients (L*, a*, and b*) of the Uruset apple vinegar were determined by a portable color measuring device (Color Measuring Device PCE-CSM-5, Spectrum Instruments, Meschede, Germany). Here L* represents luminance value, a* represents red and greenery, and b* represents yellow and blue. Before the measurement, standard white (L* = 93.71, a* = −0.84, and b* = 1.83) and black plates were used to calibrate the instrument, and the mean value was determined by repeating the color measurement tthree times for each sample.

### 2.8. Statistical Analysis

RSM (Minitab 18.1, Minitab, Inc, State College, Pensilvanya, United States) was used for the optimization of the Uruset vinegar application. Significant differences between mean values of Uruset vinegar samples were determined by an analysis of variance (one-way ANOVA) using Tukey’s HSD (honestly significant difference) test at a significance level of *p* < 0.05. Statistical analysis was conducted using SPSS 22.0 software (SPSS Inc., Chicago, IL, United States). Three-dimensional (3D) graphs of the obtained models were obtained by using SigmaPlot 12.0 Statistical Analysis Software (Systat Software, Inc., San Jose, CA, United States). All values were obtained in triplicate.

## 3. Results and Discussion

### 3.1. Total Phenolic Content and Total Flavonoid Content 

Phenolic compounds, due to their strong antioxidant properties, are effective compounds in the prevention of disease and in cancer control [14]. The equilibrium of the polynomial model—which indicates the effect of amplitude and duration on the value of the total phenolic content of Uruset vinegar samples as a result of the response surface analysis, according to the experimental design—is as follows:

TPC (mg GAE/L) = 104.8 − 9.90 *X*_1_ + 1.188 *X*_2_ − 0.4430 *X*_1_^2^ − 0.02415 *X*_2_^2^ + 0.2964 *X*_1_*X_2_ (2)

The results of the variance analysis for TPC (mg GAE/L) values of amplitude and duration on Uruset vinegar samples at different levels are given in Table 3. The model used in the study (*R*^2^ = 0.9923) was found to comply with the level (Table 3). The linear effects on the TPC values of the amplitude applied to the samples of Uruset vinegar were found to be statistically significant (*p* < 0.001). The linear effects on the TPC values of the time applied to the Uruset vinegar samples were found to be statistically significant (*p* < 0.05). Cross-interactions of amplitude and duration variables are important (*p* < 0.001).

**Figure 1:**
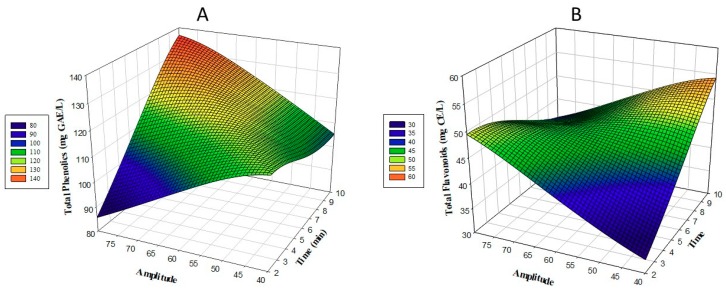
Three-dimensional (3D) response surface plots of the total phenolic content (TPC) (**A**) and total flavonoid content (TFC) (**B**) analysis, as a function of significant interaction factors.

The change in the amount of TPC, according to time and amplitude, is shown in Figure 1B. When the model was examined in terms of TPC, as the amount of time and amplitude increased a linear increase in the amount of TPC was observed. The lowest TPC value was identified for 2 min and 60% treatment in the fourth application, with the highest TPC value detected in the second application, when the sample was treated with 70% for 8 min (Table 2). The application of ultrasound to Uruset vinegar has positive effects on TPC values. At the end of optimization, TPC was determined to be 124.25 mg GAE/L after 7.4 min and 62.2 amplitude treatment (Table 6), which was a 12.5% increase compared to the C sample. Significant differences were detected between the CV (69.17 mg GAE/L) and PV (86.14 mg GAE/L) samples.

Flavonoids are natural polyphenolic compounds found in plants with a broad range of chemical and biological activities. In epidemiological studies, flavonoids were found to help reduce the risk of cardiovascular diseases and cancer. Interest in research of flavonoids from dietary sources is increasing, and it is important to evaluate the flavonoid sources in food [15]. The equilibrium of the polynomial model, indicating the effect of amplitude and duration on the TFC value of Uruset vinegar samples as a result of the response surface analysis, according to the experimental design, is as follows:

TFC (mg CE/L) = −32.44 + 10.846 × *X*_1_ + 1.3864 × *X*_2_ − 0.1121 × *X*_1_^2^ − 0.003845 × *X*_2_^2^ − 0.14359 *X*_1_*X_2_ (3)

The results of the variance analysis of TFC (mg CE/L) values, based on the amplitude and duration of Uruset vinegar treatment at different levels, are given in Table 3. It was observed that the model used in the study (*R*^2^ = 0.9931) adapted to the level (Table 3). The linear effects of amplitude and time on TFC values of Uruset vinegar samples were found to be statistically significant (*p* < 0.001). At the same time, cross-interactions of amplitude and duration variables are important (*p* < 0.001).

The change in the total amount of flavonoid, according to time and amplitude, is shown in Figure 1B. When the total amount of flavonoid was examined in the model, a linear increase in the amount of flavonoid was observed as the amount of time and amplitude increased. The lowest TFC value was found at 4 min and with 50%, in treatment number 11. The highest TFC value was detected in treatment number 1, which was treated with 50% for 8 min (Table 2). The application of the ultrasound process to Uruset vinegar has positive effects on total flavonoid values. At the end of optimization, the total flavonoid content was determined as 46.95 mg CE/L, with 7.4 min and 62.2 amplitude treatment (Table 6). There is an increase of 8.1% compared to the C sample. Significant differences were detected between CV (11.58 mg CE/L) and PV (24.12 mg CE/L) samples. It was found that the total phenolic content of ultrasound plus UV–C-treated fruits and vegetable juices increased with the checks immediately after the treatment [16]. The ultrasound treatment applied to Kasturi lime and Chokanan mango juice was found to increase phenolic and flavonoid content [17,18]. In the study, the increase in phenolic and flavonoid amount after the ultrasound process can be attributed to the release of phenolic contents due to the breakage of cell walls with cavitation pressure. Furthermore, the hydroxyl radicals produced by sonication (OH^−^) can be explained by the addition of phenolic compounds to the aromatic ring [19]. In this context, the results of the study are consistent with the literature; observed differences are due to cavitation resulting from ultrasound.

It is reported that different production methods affect the total phenolic substances and antioxidant activity values of vinegars [20]. In this study, the total amount of phenolic compounds, total amount of flavonoids, and total amount of antioxidants (DPPH and CUPRAC) were higher in Uruset vinegar than in CV and PV (Table 2).

### 3.2. DPPH and CUPRAC (Cupric Reducing Antioxidant Capacity)

Antioxidants prevent the oxidation of oxidizing compounds and play a role in reducing the risk of bacterial, cancer, and cardiovascular diseases in the body. Antioxidant agents have strong antioxidant properties, compared to free radicals and reactive oxygen species that cause cancer and cardiovascular diseases [21]. The equilibrium of the polynomial model indicating the effect of amplitude and duration on the DPPH values of Uruset vinegar samples is as follows:

DPPH (mg TEAC/mL) = 0.0335 − 0.00312 *X*_1_ + 0.015789 *X*_2_ −0.004379 *X*_1_^2^ − 0,000179 *X*_2_^2^ +0.000923 *X*_1_*X_2_ (4)

The results of variance analysis from DPPH (mg TEAC/mL) values of amplitude and duration of Uruset vinegar samples at different levels are given in Table 4. The model used in the study (*R*^2^ = 0.9931) was found to comply with these levels (Table 4). The linear effects of the time applied to Uruset vinegar samples on DPPH values were not statistically significant (*p* > 0.05). The linear effects of amplitude on the DPPH values were found to be statistically significant (*p* < 0.001). Cross-interactions of amplitude and duration variables are important (*p* < 0.001).

**Figure 2:**
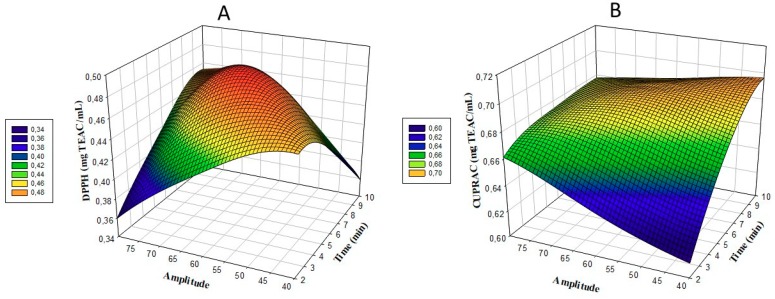
Response surface plots (3D) of DPPH (**A**) and CUPRAC (**B**) analysis as a function of significant interaction factors.

The change in the amount of DPPH in Uruset vinegar according to the time and amplitude is shown in Figure 2A. When the model was examined based on DPPH, a linear fluctuation effect was observed in DPPH amounts as time and amplitude amount increased. The lowest DPPH (mg TEAC/mL) value was obtained with 10 min and 60% amplitude treatment in treatment number 8; the highest DPPH value was detected in treatment application 3, which was treated with 60% for 6 min (Table 2). The application of ultrasound to Uruset vinegar showed positive effects on DPPH values. At the end of optimization, DPPH was found to be 0.485 (mg TEAC/mL) with 7.4 min and 62.2 amplitude treatment (Table 6). There is a 7.5% increase in the amount of DPPH compared to the C example. Significant differences were detected between CV (0.134 mg mg TEAC/mL) and PV (0.415 mg mg TEAC/mL) samples (Table 2).

The equilibrium of the polynomial model, which indicates the effect of amplitude and duration on the CUPRAC value of Uruset vinegar samples as a result of the response surface analysis, according to the experimental design, is as follows:

CUPRAC (mg TEAC/mL) = 0.3278 + 0.04675 *X*_1_ + 0,006497 *X*_2_ − 0,001708 *X*_1_^2^ − 0.000036 *X*_2_^2^ − 0.000329 *X*_1_*X_2_ (5)

The results of the variance analysis of CUPRAC (mg TEAC/mL) values for amplitude and duration on Uruset vinegar samples at different levels are given in Table 4. The model used in the study (*R*^2^ = 0.9908) was found to comply with the corresponding level (Table 4). The linear effects of the time applied to the Uruset vinegar samples on CUPRAC values were found to be statistically significant (*p* < 0.001). The linear effects of the amplitude on the CUPRAC values of the samples of Uruset vinegar were found to be statistically significant (*p* < 0.05). Cross-interactions of amplitude and duration variables are important (*p* < 0.001).

The change in the amount of CUPRAC in Uruset according to time and amplitude is shown in Figure 2B. When the CUPRAC model was examined, a linear increase effect was observed in CUPRAC amounts as time and amplitude increased. The lowest CUPRAC (mg TEAC/mL) value was obtained with 2 min and 60% amplitude, used in treatment number 4; the highest CUPRAC was detected in the number 1 treatment, treated with 50% for 8 min (Table 2). The application of ultrasound to Uruset vinegar has positive effects on CUPRAC. At the end of the optimization, CUPRAC was determined to be 0.692 (mg TEAC/mL) with 7.4 min and 62.2 amplitude treatment (Table 6). The CUPRAC amount increased by 10% according to the C example. Significant differences were found between the CV (0.213 mg mg TEAC/mL) and PV (0.586 mg mg TEAC/mL) samples (Table 2). Positive improvements in total antioxidant capacity were detected in ultrasound-treated purple cactus pear, kasturi lime, grapefruit, and carrot-grape juice [17,19,22,23]. The increase in the number of phenolic compounds as a result of cavitation induced by sonication may be considered to be in direct proportion to the total antioxidant capacity [19].

### 3.3. Color

Color is a visual indicator that is used to assess the quality of foods during processing and storage, and plays an important role in consumer satisfaction [19]. The equilibrium of the polynomial model that indicates the effect of amplitude and duration on the color values of L*, a*, and b* of Uruset vinegar samples, as a result of response surface analysis according to the experimental design, is as follows:

L* = 18.823 + 0.5655 *X*_1_ + 0.1748 *X*_2_ + 0.00201 *X*_1_^2^ − 0.001304 *X*_2_^2^ − 0.00618 *X*_1_*X_2_ (6)

a* = 29.791 − 2.1184 *X*_1_ − 0.2433 *X*_2_ − 0.00433 *X*_1_^2^ + 0.000264 *X*_2_^2^ + 0.03476 *X*_1_*X_2_ (7)

b* = −20.42 + 2.450 *X*_1_ + 0.6515 *X*_2_ − 0.11655 *X*_1_^2^ − 0.004632 *X*_2_^2^ − 0.01613 *X*_1_*X_2_ (8)

The results of analysis of variance for the color values of L*, a*, and b* of the Uruset vinegar samples for amplitude and duration, applied at different levels, are given in Table 5.

It was observed that the model used in the study conformed to the color values of L*, a*, and b*, respectively (*R*^2^ = 0.9942, 0.9917, 0.9936, respectively) (Table 5). The linear effects of L*, a*, and b* on the color of the samples applied to the Uruset vinegar samples were found to be statistically significant (*p* < 0.001). The linear effects of the amplitude on the a* and b* color values were not statistically significant (*p* > 0.05). For the L*, a*, and b* values, cross-interactions of amplitude and duration variables were found to be significant (*p* < 0.001).

**Figure 3:**
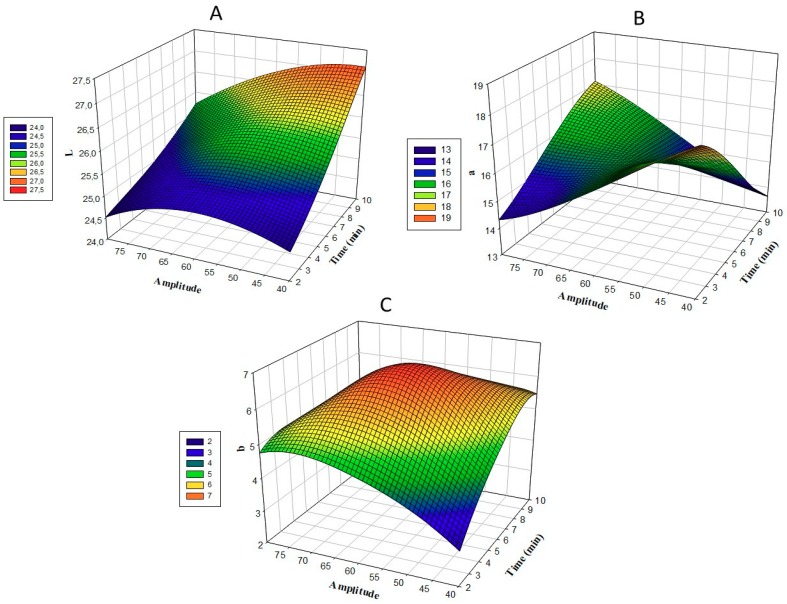
Response surface plots (3D) of L* values (**A**), a* values (**B**) and b* values (**C**) as a function of significant interaction factors.

The changes in the color values of L*, a*, and b* for Uruset vinegar, according to time and amplitude, are shown in Figure 3. When the model was examined based on DPPH, a linear fluctuation effect was observed in color values, according to the variations in the amount of time and amplitude. The lowest L* value was obtained with 6 min and 80% amplitude treatment in treatment application number 10; the highest value was detected in application 8, which was treated with 60% for 10 min (Table 2). The lowest a* value was found for 6 min and 80% amplitude treatment, in application number 10; the highest value was detected in application 8, which was treated with 60% for 10 min (Table 2). The lowest value of b* was obtained with 2 min and 60% amplitude in treatment number 2. The highest value was detected for treatment number 7, treated with 60% for 6 min (Table 2). At the end of the optimization, the values of L*, a*, and b* in the treatment with 7.4 min and 62.2 amplitude were 26.11, 15.77, and 6.51, respectively (Table 6). An increase in L* and b* values was observed with respect to the control sample, while a decrease in a* was observed (Table 2). Compared to traditional thermal protection methods, non-thermal methods are said to provide better protection for food, flavor, and color [24]. As a result of ultrasound application, it was stated that the collapse of unstable particles may be responsible for the increase in L* values [25]. It was also reported that the increase in L* value may be due to the increase in cloud value of fruit juice under the influence of sonication, resulting in homogenization [26]. It has been suggested by researchers that a decrease in the a* value is related to anthocyanin degradation and the formation of Maillard reaction products [27].

## 4. Conclusions

In this study, ultrasound-treated vinegar produced from Uruset apples, whose bioactive property is higher, was optimized using the response surface method. At the same time, the difference between the ultrasound-treated Uruset diamond vinegar and commercial apple vinegar, as well as between pasteurized Uruset apple vinegar and untreated Uruset apple vinegar were investigated. The results of the analysis found that Uruset apple vinegar with ultrasound applied and optimized was high in terms of total phenolic substance, total flavonoids, total antioxidant capacity, and color values. According to these results, the maximized percentage yield and the acidity and peroxide value were minimized among the three dependent variables. The common independent variables determined for each of the three variables were 7.4 min and 62.2 amplitudes, respectively, for the duration and amplitude. As a result, ultrasound technology was successful for Uruset apple vinegar production. In the case of industrial production of the product, optimization conditions must be increased, and microbial safety must be examined.

## Figures and Tables

**Table 1:** Independent variables and their levels in the response surface method.

	Factor Levels				
Independent variable	Lowest	Low	Center	High	Highest
	(−1.41)	(−1)	0	(+1)	(1.41)
Time (Factor 1, *X*_1_)	2	4	6	8	10
Amplitude (Factor 2, *X*_2_)	40	50	60	70	80

**Table 2:** Measured responses used in experimental design for RSM.

Sample	Encoded Independent Variables		Dependent Variables						
	Time (X_1_)	Amplitude (X_2_)	Response 1	Response 2	Response 3	Response 4	Response 5	Response 6	Response 7
			Total Phenolics Compound (mg GAE/L)	Total flavonoids (mg CE/L)	DPPH (mg TEAC/mL)	CUPRAC (mg TEAC/mL)	*L*	*a*	*b*
CV			69.17	11.58	0.134	0.213	35.60	5.18	15.48
PV			86.14	24.12	0.415	0.586	24.36	15.24	5.65
C			108.70	43.14	0.449	0.623	25.36	16.04	6.06
1	8 (+1)	50(−1)	115.26	49.443	0.438	0.692	26.48	14.93	6.19
2	8 (+1)	70 (+1)	127.62	44.650	0.473	0.686	25.85	16.24	5.54
3	6 (0)	60 (0)	121.22	46.700	0.495	0.687	25.93	15.75	6.66
4	2 (−1.41)	60 (0)	103.01	40.760	0.424	0.633	25.06	16.02	4.56
5	6 (0)	60 (0)	119.65	46.143	0.493	0.685	25.91	15.82	6.74
6	6 (0)	60 (0)	120.92	46.210	0.494	0.688	25.85	15.85	6.77
7	6 (0)	60 (0)	121.42	46.180	0.491	0.687	25.86	15.77	6.76
8	10 (+1.41)	60 (0)	123.82	48.150	0.422	0.687	26.76	15.43	5.20
9	4 (−1)	70 (+1)	105.68	47.150	0.438	0.674	25.17	15.28	5.82
10	6 (0)	80 (+1.41)	112.67	46.140	0.418	0.674	24.99	15.85	4.86
11	4 (−1)	50 (−1)	117.04	40.456	0.476	0.654	25.31	16.75	5.18
12	6 (0)	60 (0)	119.33	46.010	0.492	0.689	25.82	15.78	6.74
13	6 (0)	40 (−1.41)	109.02	43.280	0.425	0.671	25.73	15.95	4.92

PV: Pasteurized Uruset vinegar; CV: Commercial apple vinegar; GAE: Gallic acid equivalent; DDPH: radical scavenging activity; CUPRAC: Cupric Reducing Antioxidant Capacity; L*: represents luminance value a*: represents red and greenery; b*: represents yellow and blue.

**Table 3:** Analysis of variance (ANOVA) of responses for total phenolic and total flavonoid experiments.

Source	Total Phenolics Compound (mg GAE/L)					Total Flavonoids (mg CE/L)			
	DF	SS ^1^	MS	*F*-Value	*p*-Value	SS ^1^	MS	*F*-Value	*p*-Value
Model	5	627.396	125.479	180.33	0.0000	817.695	163.539	201.73	0.0000
Linear	2	323.797	161.898	232.67	0.0000	425.301	21.265	262.31	0.0000
*X*_1_	1	318.069	318.069	457.11	0.0000	376.899	376.899	464.91	0.0000
*X*_2_	1	5.728	5.728	8.23	0.024	48.402	48.402	59.7	0.0001
Square	2	163.005	81.503	117.13	0.0000	62.525	31.262	38.56	0.0002
*X*_1_*X_1_	1	71.957	71.957	103.41	0.0000	46.037	46.037	56.79	0.0001
*X*_2_*X_2_	1	133.584	133.584	191.98	0.0000	33.873	33.873	41.78	0.0003
2-Way Interaction	1	140.594	140.594	202.05	0.0000	32.987	32.987	406.9	0.0000
*X*_1_*X_2_	1	140.594	140.594	202.05	0.0000	32.987	32.987	406.9	0.0000
Error	7	4.871	0.696			0.5675	0.0811		
Lack-of-Fit	3	1.247	0.416	0.46	0.7258	0.2894	0.0965	1.39	0.3681
Pure Error	4	3.624	0.906			0.2781	0.0695		
Total	12	632.267				82.337			
*R* ^2^		0.9923				0.9931			
Adj *R*^2^		0.9868				0.9882			
Pred *R*^2^		0.9716				0.9593			

^1^ Sum of squares; DF: degree of freedom; MD: mean squares *: multiplication. The term is significant at *p* ≤ 0.05. The term is significant at *p* ≤ 0.01. The term is significant at *p* ≤ 0.001.

**Table 4:** Analysis of variance (ANOVA) of responses for 1,1-diphenyl- 2-picrylhydrazyl (DPPH) and cupric reducing antioxidant capacity (CUPRAC) experiments.

Source	DPPH (mg TEAC/mL)					CUPRAC (mg TEAC/mL)			
	DF	S ^1^	MS	*F*-Value	*p*-Value	SS ^1^	MS	*F*-Value	*p*-Value
Model	5	0.01258	0.002516	1041.04	0.0000	0.003383	0.000677	150.16	0.0000
Linear	2	0.000028	0.000014	5.77	0.0331	0.002069	0.001035	229.59	0.0000
*X*_1_	1	0.000004	0.000004	1.65	0.24	0.00204	0.00204	452.67	0.0000
*X*_2_	1	0.000024	0.000024	9.89	0.0163	0.000029	0.000029	6.51	0.038
Square	2	0.011188	0.005594	2314.77	0.0000	0.001141	0.00057	126.59	0.0000
*X*_1_*X_1_	1	0.00703	0.00703	2909.06	0.0000	0.001069	0.001069	237.2	0.0000
*X*_2_*X_2_	1	0.007334	0.007334	3034.53	0.0000	0.000303	0.000303	67.33	0.0001
Two-way Interaction	1	0.001363	0.001363	564.1	0.0000	0.000173	0.000173	38.43	0.0004
*X*_1_*X_2_	1	0.001363	0.001363	564.1	0.0000	0.000173	0.000173	38.43	0.0004
Error	7	0.000017	0.000002			0.000032	0.000005		
Lack-of-Fit	3	0.000007	0.000002	0.86	0.5306	0.000022	0.000007	3.11	0.1506
Pure Error	4	0.00001	0.000003			0.000009	0.000002		
Total	12	0.012597				0.003415			
*R* ^2^		0.9987				0.9908			
Adj *R*^2^		0.9987				0.9842			
Pred *R*^2^		0.9944				0.9302			

^1^ Sum of squares; DF: degree of freedom; MD: mean squares *: multiplication. The term is significant at *p* ≤ 0.05. The term is significant at *p* ≤ 0.01. The term is significant at *p* ≤ 0.001.

**Table 5:** Analysis of variance (ANOVA) of responses for L*, a*, and b* experiments.

Source	L*					a*				b*			
	DF	SS ^1^	MS	*F*-Value	*p-*Value	SS ^1^	MS	*F*-Value	*p-*Value	SS ^1^	MS	*F*-Value	*p-*Value
Model	5	3.22643	0.64529	241.98	0	2.32207	0.46441	168.28	0	8.45916	1.69183	217.92	0
Linear	2	2.72531	1.36265	510.98	0	0.35788	0.17894	64.84	0	0.33535	0.16768	21.6	0.001
*X*_1_	1	2.30493	2.30493	864.33	0	0.34711	0.34711	125.78	0	0.33403	0.33403	43.02	0.0003
*X*_2_	1	0.42038	0.42038	157.64	0	0.01077	0.01077	3.9	0.0888	0.00132	0.00132	0.17	0.6923
Square	2	0.44012	0.22006	82.52	0	0.0314	0.0157	5.69	0.0341	7.70772	3.85386	496.39	0
*X*_1_*X_1_	1	0.00148	0.00148	0.56	0.48	0.00688	0.00688	2.49	0.1583	4.98007	4.98007	641.45	0
*X*_2_*X_2_	1	0.38949	0.38949	146.06	0	0.01602	0.01602	5.81	0.0468	4.91618	4.91618	633.22	0
Two-way Interaction	1	0.06101	0.06101	22.88	0.002	1.9328	1.9328	700.36	0	0.41609	0.41609	53.59	0.0002
*X*_1_*X_2_	1	0.06101	0.06101	22.88	0.002	1.9328	1.9328	700.36	0	0.41609	0.41609	53.59	0.0002
Error	7	0.01867	0.00267			0.01932	0.00276			0.05435	0.00776		
Lack-of-Fit	3	0.01109	0.0037	1.95	0.2635	0.0128	0.00427	2.62	0.1878	0.04683	0.01561	8.3	0.0342
Pure Error	4	0.00758	0.00189			0.00652	0.00163			0.00752	0.00188		
Total	12	3.2451				2.34139				8.51351			
*R* ^2^		0.9942				0.9917				0.9936			
Adj *R*^2^		0.9901				0.9859				0.9891			
Pred *R*^2^		0.9692				0.9403				0.9604			

^1^ Sum of squares; DF: degree of freedom; MD: mean squares; *: multiplication. The term is significant at *p* ≤ 0.05. The term is significant at *p* ≤ 0.01. The term is significant at *p* ≤ 0.001.

**Table 6:** Maximum optimization values, according to the response surface method.

Variable	Setting			
*X*_1_	7.4			
*X*_2_	62.2			
Response	Fit	SE Fit	95% CI	95% PI
b*	6.51	0.04	(6.4196; 6.5960)	(6.2816; 6.7341)
a*	15.77	0.02	(15.7154; 15.8206)	(15.6331; 15.9029)
L*	26.11	0.02	(26.0537; 26.1571)	(25.9728; 26.2380)
CUPRAC (mg TEAC/mL)	0.69	0.00	(0.690020; 0.694270)	(0.686694; 0.697596)
DPPH (mg TEAC/mL)	0.49	0.00	(0.483650; 0.486761)	(0.481214; 0.489197)
Total Flavonoids (mg CE/L)	46.95	0.12	(46.666; 47.236)	(46.220; 47.682)
Total Phenolics (mg GAE/L)	124.25	0.35	(123.416; 125.086)	(122.109; 126.393)
